# Spatial patterns of dominant bacterial community components and their influential factors in the southern Qinling Mountains, China

**DOI:** 10.3389/fmicb.2022.1024236

**Published:** 2022-12-23

**Authors:** Yonghua Zhao, Manya Luo, Yujie Zhou, Xia Jia, Shuaizhi Kang, Shuyuan Yang, Qi Mu

**Affiliations:** Key Laboratory of Degraded and Unused Land Consolidation Engineering of the Ministry of Natural Resources, Shaanxi Key Laboratory of Land Consolidation, School of Land Engineering, School of Water and Environment, Shaanxi Provincial Land Consolidation Engineering Technology Research Center, Chang’an University, Xi’an, China

**Keywords:** bacterial phyla, spatial pattern, topographic factors, environmental factors, soil nutrients, Qinling Mountains

## Abstract

**Introduction:**

Soil bacteria not only maintain the biodiversity of forest ecosystems but also affect soil nutrient cycling and ecosystem function. Nonetheless, the spatial pattern and patchy distribution of dominant bacterial community components in soil are still rarely explored.

**Method:**

The spatial pattern and distribution of the dominant bacterial community components and their influential factors were investigated using traditional statistics, geostatistics, and kriging spatial interpolation methods in the Huoditang region of the Qinling Mountains, China.

**Results:**

The dominant bacterial phyla were Proteobacteria, Acidobacteria, Chloroflexi, Rokubacteria, Actinobacteria, and Verrucomicrobia in this region. Among the bacterial phyla, Proteobacteria occupied an area of 2.56 km^2^ (the greatest) in the highest patch category, followed by Planctomycetes. Moreover, among the lowest patch category, Firmicutes occupied the lowest area (11.93 km^2^). The results of kriging maps showed that the dominant bacterial group presented “peak,” “bimodal,” and “multimodal” distributions in Huoditang. Proteobacteria, Acidobacteria, Chloroflexi, Gemmatimonadetes, Nitrospirae, and ASV (amplicon sequence variants) had significant spatial autocorrelation (< 0.68 km). Variance partitioning analysis confirmed that soil nutrients (36.5%) were the significant driving factors shaping the bacterial community structure, followed by environmental factors (28.2%) and topographic factors (7.8%). Furthermore, pH (9.1%), soil organic carbon (SOC, 6.6%), available phosphorus (AP, 4.7%), and elevation (3.9%) were the most important driving factors for the spatial distribution of bacterial community groups in the Huoditang Forest of the Qinling Mountains. The findings provide a new perspective for studying the spatial distribution characteristics and driving factors of dominant soil bacterial community components in subtropical forest ecosystems.

## Introduction

Soil bacteria play a crucial role in nutrient cycling and ecosystem stability (Fierer, 2017). Previous studies have shown that spatial factors can explain the changes in soil bacterial community composition better than environmental characteristics (Liu et al., 2018; Song et al., 2018; Kang et al., 2021). Therefore, clarifying the spatial pattern of the soil bacterial community is of great significance for a better understanding of the potential mechanisms, functional stability, and nutrient cycling of forest ecosystems (Naveed et al., 2016; Li et al., 2019; Liu et al., 2020).

In subtropical forests, it is necessary to facilitate the interaction and model fitting between soil characteristics and the bacterial community structure. In fact, the spatial heterogeneity of bacterial community component is likely to be dominated by potential abiotic and biological factors such as elevation (Siles and Margesin, 2016; Tang et al., 2020), soil properties, and the normalized difference vegetation index (NDVI; Shen et al., 2013, 2020; Zeng et al., 2019; Zhao et al., 2020). An increasing number of studies have demonstrated significant soil nutrient spatial heterogeneity and patterns on different regional scales (Liu et al., 2013), which will not only affect terrestrial biological carbon and nitrogen cycles (Hoffmann et al., 2014; Yao et al., 2019) but will also cause significant changes in the structure and diversity of microbial communities through plant growth and litter decomposition (Lauber et al., 2008; Rodríguez et al., 2011; Song et al., 2018; Zhao et al., 2018). For example, Liu et al. (2014) found that the spatial distribution of the soil bacterial community composition and diversity was mainly affected by soil pH and the soil total carbon content in the black soil region of northeastern China. A study of alpine wetlands confirmed that spatial structure explained 30% of the variation in the bacterial community structure in different soil layers, which was mainly driven by pH and soil nutrients (Kang et al., 2021). Despite the differences in the distribution patterns of bacterial communities due to spatial variations such as latitude (Liu et al., 2014; Singh et al., 2014), studies on the spatial distribution of small-scale bacterial communities and their driving factors are still limited. Alternatively, how to quantify the relative contribution of different drivers (e.g., soil nutrients, topography, and environmental factors) to spatial patterns remains to be explored, limiting our understanding of the mechanism by which microorganisms influence several nutrient cycles in land ecosystems.

The Huoditang Forest, a representative observation station in the Qinling Mountains, which is the geographical boundary between northern and southern China, has a typical subtropical climate, a representative vegetation type, and a complete ecological observation system (Yuan et al., 2018). Previous studies have investigated microbial distribution patterns at different geographic scales such as elevation (Shen et al., 2020), drought (Jiao and Lu, 2020), and latitudinal gradients (Zheng et al., 2021). Whether dominant bacterial taxa have specific spatial distribution patterns remains unknown, especially in forest ecosystems. There are no detailed investigations on the spatial heterogeneity of soil bacterial communities and their driving factors (i.e., topographic factors and soil characteristics) in mountain ecosystems. This limits our ability to predict and understand the potential ecological functions and the spatial patterns of soil microbial diversity in subtropical forests. To explore the spatial distribution characteristics and driving forces of dominant bacterial taxa. Here, amplicon sequencing (16S rRNA gene), kriging interpolation, geostatistics, and conventional statistical methods were used to investigate soil bacterial taxa at the phylum (> 1%) and ASV levels. The objectives of this study were: (1) to investigate the dominant community components and spatial heterogeneity of the bacterial community in the Huoditang forest, Qinling Mountains, and (2) to analyze the influential factors of dominant bacterial community components. Our results will further promote the understanding of the spatial patterns of dominant soil bacterial community components and their responses to soil characteristics.

## Materials and methods

### Study site and sampling

This study was conducted in the Huoditang Forest (~ 22 km^2^) in the southeastern part of the Qinling Mountains (33°17′–33°19′N, 108°20′–108°41′E), which are located in Ningshan County, Ankang city, Shaanxi Province, China. The sampling site is characterized by a subtropical climate, with an average annual temperature (MAT) of 12.7°C and mean annual precipitation (MAP) of 1,000 mm (July–August), at an altitude of 1,500–2,400 m above the sea level. The vegetation coverage of the Huoditang Forest has reached 91.8%, which is mainly coniferous forest, deciduous broad-leaved forest, and evergreen broad-leaved mixed forest. Due to extensive logging in the last century, the native vegetation in the study area has been replaced by secondary vegetation. There are many dominant tree species, such as *Pinus armandii* Franch, *Pinus tabuliformis* Carr, *Carpinus turczaninovii*, *R. omeiensis*, and *Quercus aliena.* Var. *acuteserrata*, *Lonicera tragophylla*, *Tsuga chinensis*, and Betula.

From July to September 2018, a total of 199 sampling sites (30 × 30 m) with a horizontal distance greater than 300 m were established according to the grid method along an altitude of 1,500–2,400 m in the Huoditang Forest region of the Qinling Mountains; thus, each site was considered independent of each other. After the removal of the litter and branches on the soil surface, three soil samples of 10–20 cm were collected using a 55 mm diameter soil sampler and mixed into a composite sample of 1800*g* at each site, resulting in a total of 199 samples. The elevation, slope, and aspect of each site were simultaneously recorded in the field using a hand-held GPS (GPSMAP 639CSX, Garmin) and compass as topographic factors. The collected soil samples were divided into two parts; one was stored at room temperature for analysis of the soil physical and chemical properties, and the other was stored in a refrigerator at −80°C for soil DNA extraction.

### Environmental and physico-chemical characterization of the soil samples

Soil temperature (ST) and soil moisture (SM) were determined in the field by a time-domain reflectometry (TDR) soil multiparameter monitoring system (AIM-WIFI TDR, Germany). Soil pH was measured using a pH meter (Mettler-Toledo S220, Switzerland) with a soil-to-water ratio (*w*/*v*) mixture of 1:2.5 by the addition of a CO_2_-free distilled water suspension for 30 min (Zhou et al., 2021). SOC, the carbon-to-nitrogen ratio (C/N), AP, and available potassium (AK) were used as soil nutrients (Li et al., 2020). SOC and the C/N ratio were analyzed with an elemental analyzer (Vario Macro cube, Elementar, Hanau, Germany). Available phosphorus (AP) in agreement with the study described by Zhou et al. (2021), which was measured by colorimetric analysis of NaHCO_3_ soil extracts. (Nu, 1999). Moreover, available potassium (AK) was measured by the flame photometry method according to Pansu and Gautheyrou (2006).

In this study, the normalized difference vegetation index (NDVI) was used to represent vegetation factors. The NDVI was calculated using Sentinel-2 remote sensing image data,^1^ which were the 10-meter spatial resolution data from the summer of 2019. The L2A level data were generated by using the Sen2Cor plug-in to perform radiation calibration and atmospheric correction of the L1C level data. Sentinel-2 band 4 (red in a range of 650–680 nm) and band 8 (NIR in a range of 785–900 nm) were used for the calculation of NDVI.

### DNA extraction, amplification, and high-throughput sequencing

Soil genomic DNA was extracted from 0.5 g fresh soil using a Fast DNA Spin Kit (Omega, Norcross, Georgia, United States) and stored at −20°C prior to further analysis. The quantity and quality of extracted DNA were measured using a NanoDrop ND-1000 spectrophotometer (Thermo Fisher Scientific, Waltham, MA, United States) and agarose gel electrophoresis, respectively.

The V3–V4 variable regions of the 16S rRNA gene were amplified using the universal primers 338F (5′-ACTCCTACGGGAGGCAGCA-3′) and 806R (5′-GGACTACHVGGGTWTCTAAT-3′). The PCRs were performed using the following procedure: denaturation at 98°C for 2 min, followed by 25 cycles of denaturation at 98°C for 15 s, annealing at 55°C for 30 s, and extension at 72°C for 30 s, with a final extension step at 72°C for 5 min. The amplification quality was checked and purified by 2% agarose gel electrophoresis and purified using an Axygen DNA gel Extraction Kit (Axygen Biosciences, Union City, CA, United States). Finally, purified amplicons were sequenced (2 × 300 bp) on an Illumina MiSeq platform with MiSeq Reagent Kit v3 (600 cycles) at Shanghai Personalbio Technology Co., Ltd. (Shanghai, China).

### Sequence analysis

Paired-end reads were assembled to obtain raw tags using FLASH (Version 1.2.11; Magoc and Salzberg, 2011).^2^ The quantitative insights into microbial ecology (Quantitative Insights into Microbial Ecology, QIIME 2)^3^ pipeline was employed to process the sequencing data. Sequences of poor quality (length < 150 bp, Phred quality score < 15) were removed from the dataset. The dada 2 method was used for depriming, filtering, and denoising based on QIIME2 software (v 2019.4; Callahan et al., 2016). An amplicon sequence variants (ASVs) feature table was generated based on dereplicated sequences with 100% similarity. ASVs taxonomic classification was performed by searching the representative sequence set against the Silva database (Release 132)^4^ using the best hit (Quast et al., 2013).

### Statistical analyses

The geostatistics method explores spatial distribution and heterogeneity by establishing a probability model of spatial continuity through specific sampling data (Zhao et al., 2009). The coefficient of variation (CV) was used to compare the degree of dispersion between the data. The semivariogram was used to select the best model (i.e., Gaussian, spherical, linear, and exponential) through the regression value (*R*^2^, maximum) and residual sums of squares (RSS, minimum) in GS+ 7.0 software. Taxa data were transformed by the centered log-ratio (clr) to ensure the accuracy and reliability of the model (Aitchison, 1986; Gloor et al., 2017). The semivariogram model can be divided into three parameters, the nugget variance (C_0_), still (C_1_ + C_0_), and degree of spatial dependence (GD), which can identify the spatial structure of the nugget variable at a certain scale (Griffith and Layne, 1999). The best-fitting model was used to predict the spatial distribution of the soil bacterial community structure (Song et al., 2018). The ordinary kriging interpolation method was used to predict the spatial distribution through the weighted average of samples (Zhou et al., 2021). A spatial distribution map was produced in ArcGIS. Moran’s *I* index was used to assess whether there was a dependency on the spatial distribution of the bacterial community structure (Song et al., 2018; Peng et al., 2019). The semivariogram for the soil bacterial community is calculated as follows:


$$\gamma \left(h\right)=\frac{1}{2N\left(h\right)}\overset{N\left(h\right)}{\underset{i=1}{\sum }}[Z{\left(X_{i}-Z\left(X_{i+h}\right)\right]}^{2},$$

where *h* represents lag distance, *N*(*h*) represents the number of sample points separated by *h*, and *z*(*x_i_*) and *z*(*x_i + h_*) are the investigated fungal taxa in two places.

Based on Sentinel-2 L2A level data, NDVI was calculated by band math using ENVI 5.1 software (Exelis Visual Information Solutions, America). Spearman’s correlation analysis was used to analyze the correlation between bacterial taxa and NDVI, soil nutrients, environmental factors, and topographic factors. Data correlation was calculated by Spearman’s rank method in SPSS 22.0 and visualized using the Matplotlib package in Python.^5^ Variance decomposition analysis (VPA) was conducted to appraise the relative importance and explanatory rates of topographical factors, soil nutrients, and environmental factors for the bacterial community structure by Canoco 5.0 software. Redundancy analysis (RDA) was used to disentangle the links between driving factors (topography, environment, and nutrients) and the bacterial community structure and to explore the relative importance of each factor internally using Canoco 5.0.

## Results

### Soil characteristics

The soil characteristic status of the Huoditang Forest region is shown in Table 1. The vegetation factor (NDVI) showed a low coefficient of variation (CV < 25%), ranging from 0.29 to 0.91 (mean = 0.86). Among the topographic factors, elevation (mean = 1913.15 m) presented the lowest variability (CV = 10.85%), whereas slope (mean = 23.65°) and aspect displayed moderate variations of 42.47 and 45.47%, respectively. There was low variation (CV < 25%) in environmental factors including soil pH, SM, and ST, and the values ranged from 5.18 to 7.98 (mean = 6.62), 10.10 to 43.29% (mean = 28.16) and 15.9 to 32.0°C (mean = 23.36), respectively. The overall SOC, AP, AK, and C/N contents fluctuated more (CV > 50%), with ranges of 16.27–151.3, 0.21–36.0, 0.7–679.6, and 6.18–31.98 mg kg^−1^, respectively (Table 1).

**Table 1:** Descriptive statistics of variable of vegetation factor, topographic factors, environmental factors, and soil nutrients.

Factor type	Variables	Maximum	Minimum	Mean	SE	CV (%)	DT
Vegetation factor	NDVI	0.91	0.29	0.86	0.076	8.84	LN
Topographical factors	Elevation (m)	2367.00	1518.00	1913.15	207.58	10.85	N
	Slope (°)	45	5	23.65	14.85	42.47	N
	Aspect (°)	345.96	7.24	188.24	85.59	45.47	N
Environmental factors	pH	7.98	5.18	6.62	0.66	9.90	N
	ST (°C)	32.0	15.90	23.36	5.05	21.6	LN
	SM (%)	43.29	10.10	28.16	6.94	24.60	LN
Soil nutrients	AP (mg kg^−1^)	36.00	0.21	12.34	9.24	74.90	N
	SOC (g kg^−1^)	151.3	16.27	40.98	21.9	53.44	LN
	AK (mg kg^−1^)	679.60	0.70	161.59	125.52	77.60	LN
	C/N	31.98	6.18	11.56	2.56	22.20	LN

NDVI, normalized difference vegetation index; SM, soil moisture; ST, soil temperature; SOC, soil organic carbon; C/N, soil C:N ratio; AP, available phosphorus; AK, available potassium; DT, distribution type; N, normal distribution; LN, log-normal distribution.

### Soil bacterial community taxonomic composition

**Figure 1:**
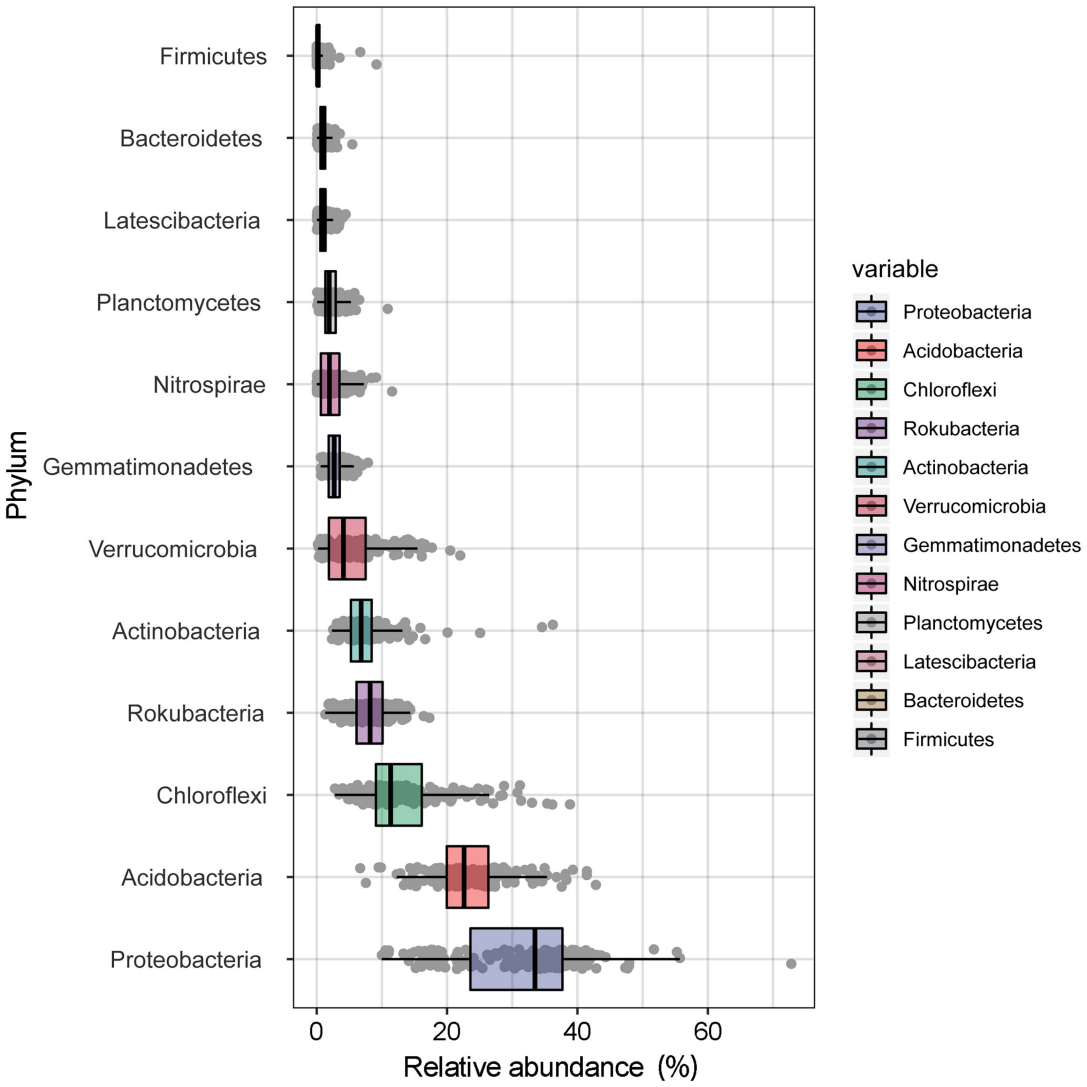
The average relative abundance of the dominant bacteria at phyla level in the Huoditang Forest soils in Qinling Mountains, China.

A total of 9,470,157 (mean = 47,589) high-quality sequences were obtained from 199 samples. Twelve bacterial phyla were observed, including Proteobacteria (31.28%), Acidobacteria (23.52%), Chloroflexi (13.39%), Rokubacteria (8.17%), Actinobacteria (7.47%), Verrucomicrobia (5.36%), Gemmatimonadetes (2.88%), Nitrospirae (2.31%), Planctomycetes (2.25%), Latescibacteria (1.09%), Bacteroidetes (1.05%), and Firmicutes (0.43%; Figure 1). The most dominant bacterial phyla tended to be moderately variable, with CV values ranging from 25 to 75%, except for Acidobacteria (< 25%) and Nitrospirae (> 75%; Supplementary Table S1). Among the dominant bacterial community components, Proteobacteria, with the highest abundance, occupied the largest patch area (2.56 km^2^), while Firmicutes occupied the smallest area (0.001 km^2^), the elevation ranges were 1,570–2018 m and 1784–1815 m, respectively (Table 2).

**Table 2:** The spatial patterns of the abundance of the dominant bacteria and amplicon sequence variants (ASVs) at phylum level.

Species	Patch categories	Patch area (km^2^)	Elevation range (m)
Proteobacteria	Max	2.56	1,570–2018
	Min	0.55	2094–2,330
Acidobacteria	Max	0.47	2011–2,318
	Min	1.70	1,595–4,591
Chloroflexi	Max	0.6	1945–2,188
	Min	2.88	1,618–1911
Rokubacteria	Max	0.005	2,214–2,301
	Min	0.05	1765–1799
Actinobacteria	Max	0.14	2,109–2,274
	Min	4.63	2007–2,305
Verrucomicrobia	Max	0.72	2,284–2,323
	Min	0.14	1850–1896
Gemmatimonadetes	Max	0.198	1,577–1,628
	Min	2.49	2,132–2,287
Nitrospirae	Max	0.14	1,604–1,679
	Min	1.85	1815–2,259
Planctomycetes	Max	1.04	1780–2,328
	Min	0.23	1856–1906
Latescibacteria	Max	0.47	2,257–2,356
	Min	1.94	1,513–1789
Bacteroidetes	Max	0.002	1925–1988
	Min	2.68	1,604–2,304
Firmicutes	Max	0.001	1784–1815
	Min	11.93	1,508–2,325
ASVs/OTUs	Max	4.82	1905–2,159
	Min	9.77	1,577–2012

Max represents maximum, Min represents minimum.

### Spatial patterns of the dominant groups

**Figure 2:**
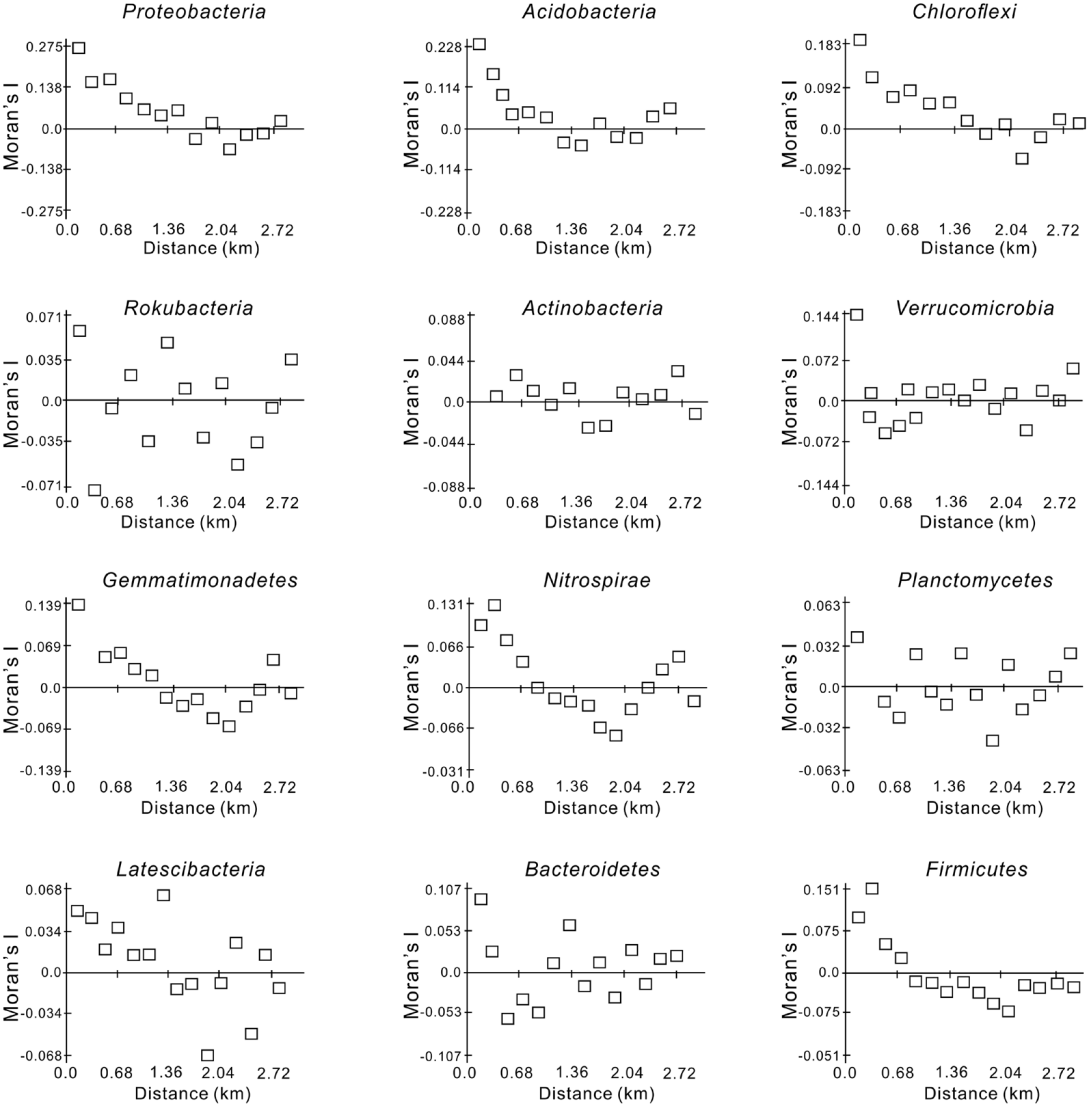
Moran’s*I*index of the dominant soil bacterial phyla in the Huoditang Forest in Qinling Mountains, China.

The spatial autocorrelation Moran’s *I* index for the dominant bacterial phyla is shown in Figure 2. Proteobacteria, Acidobacteria, Chloroflexi, Gemmatimonadetes, and Nitrospirae presented obvious regularity and similar spatial structures, showing a “U”-shaped pattern with increasing lag distance and reaching a minimum at 2.04 km (Figure 2). The comparatively high Moran’s *I* values ranging from −0.092 to 0.275 indicated that the six dominant phyla were relatively distinct in their dependence on the spatial structure. The Moran’s *I* indices of Actinobacteria and Verrucomicrobia were close to 0 at closer distances (< 0.3 km), and the range of variation was small. Moreover, the Moran’s *I* indices of the other four bacterial phyla (Rokubacteria, Planctomycetes, Latescibacteria, and Bacteroidetes) showed no obvious regularity and alternated between positive and negative correlations. The variation was relatively small between −0.071 and 0.105 and close to 0 at approximately 0.6 km (Figure 2).

Based on the maximum regression coefficient (*R*^2^) and the minimum RSS (residual sums of squares), the geostatistical and semivariogram fitting models of the dominant phylum were selected, as shown in Table 3. The exponential model was fitted for Proteobacteria, Acidobacteria, Chloroflexi, and Firmicutes, while the spherical model was more suitable for Rokubacteria, Actinobacteria, Verrucomicrobia, and Bacteroidetes. Moreover, the dominant phyla Gemmatimonadetes, Nitrospirae, and Latescibacteria were fit to a linear model, and a Gaussian model was suitable for Planctomycetes. The *C*/(*C*_o_ + *C*) value in the dominant phylum Bacteroidetes was within 25–75%, showing moderate spatial dependence. In addition, the dominant bacterial phyla showed strong spatial dependence (< 25%), except for Gemmatimonadetes and Latescibacteria (> 75%).

**Table 3:** Geostatistical parameters on the relative abundance of bacteria at phylum and ASV/OTU level.

Species	Model	C_0_	Still (c_0_ + c_1_)	A_0_ (m)	GD (%)	R^2^	RSS
Proteobacteria	Exponential	0.0117	0.1214	450	9.64	0.633	1.24E-03
Acidobacteria	Exponential	0.0001	0.0036	362	2.78	0.629	2.45E-07
Chloroflexi	Exponential	0.0215	0.227	298	9.47	0.52	1.34E-03
Rokubacteria	Spherical	0.0002	0.003	232	6.67	0.188	2.76E-07
Actinobacteria	Spherical	0.0092	0.1904	324	4.83	0.649	1.28E-03
Verrucomicrobia	Spherical	0.0001	0.002	277	5	0.558	9.88E-08
Gemmatimonadetes	Linear	0.00016	0.00018	3,406	88.89	0.236	1.29E-09
Nitrospirae	Linear	0.00003	0.0002	3,406	15	0.767	3.26E-09
Planctomycetes	Gaussian	0.00003	0.0002	106	15	0.049	2.52E-09
Latescibacteria	Linear	0.00006	0.00007	3,406	85.71	0.78	9.83E-11
Bacteroidetes	Spherical	0.00002	0.00005	385	40	0.582	1.08E-10
Firmicutes	Exponential	0.00002	0.0001	163	20	0.308	2.16E-09
ASVs/OTUs	Exponential	0.00003	0.0002	223	11.24	0.781	3.12E-09

*C*_0_ represents nugget variance, *A*_0_ represents lag distance, *C*_1_ represents structure of variance, *C*_0_ + *C*_1_ represents the sill variance, GD represents the degree of spatial dependence, *R*^2^ represents regression value, and RSS represents residual sums of squares.

**Figure 3:**
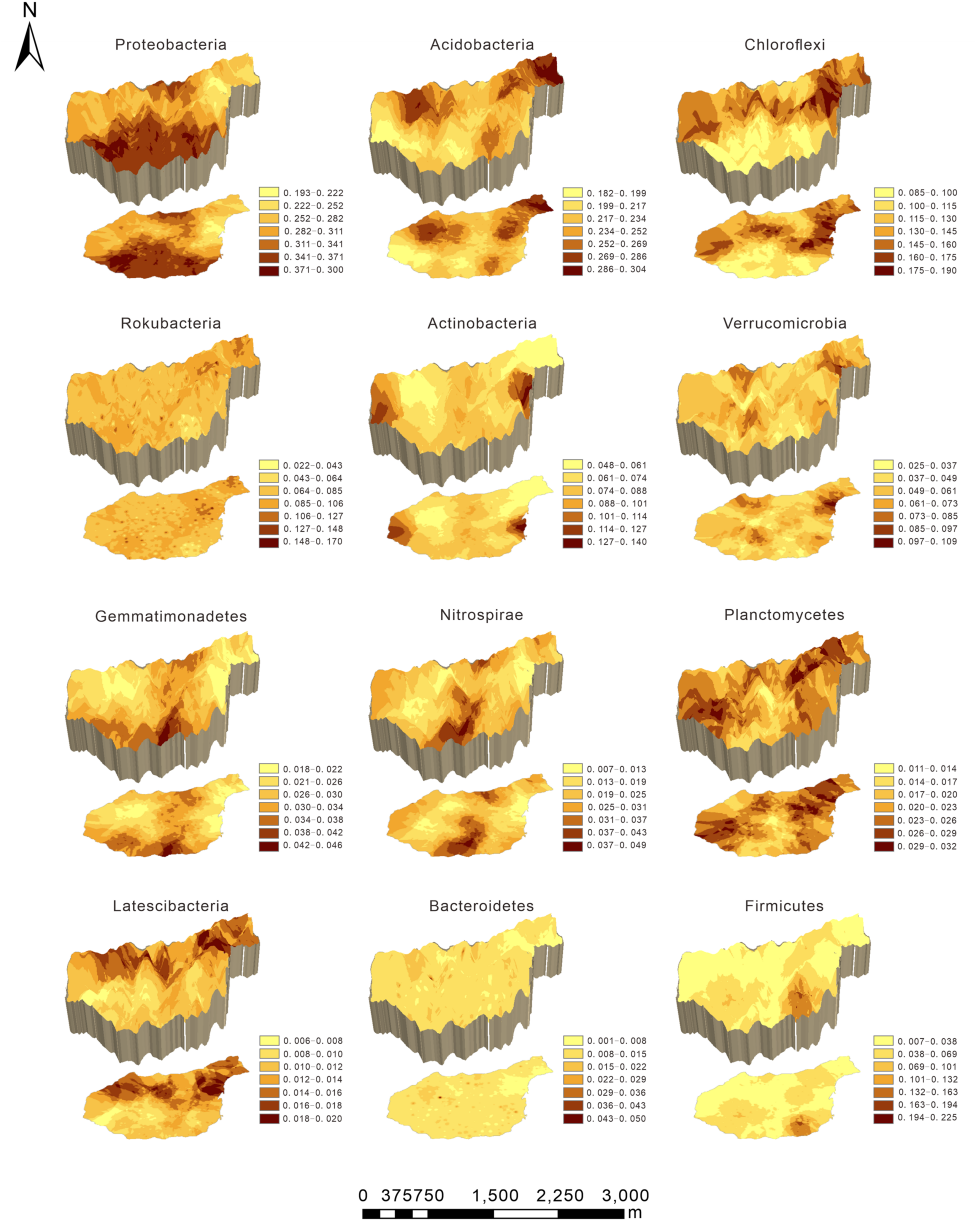
Spatial characteristics of the dominant bacterial phyla in the Huoditang Forest in Qinling Mountains, China.

**Figure 4:**
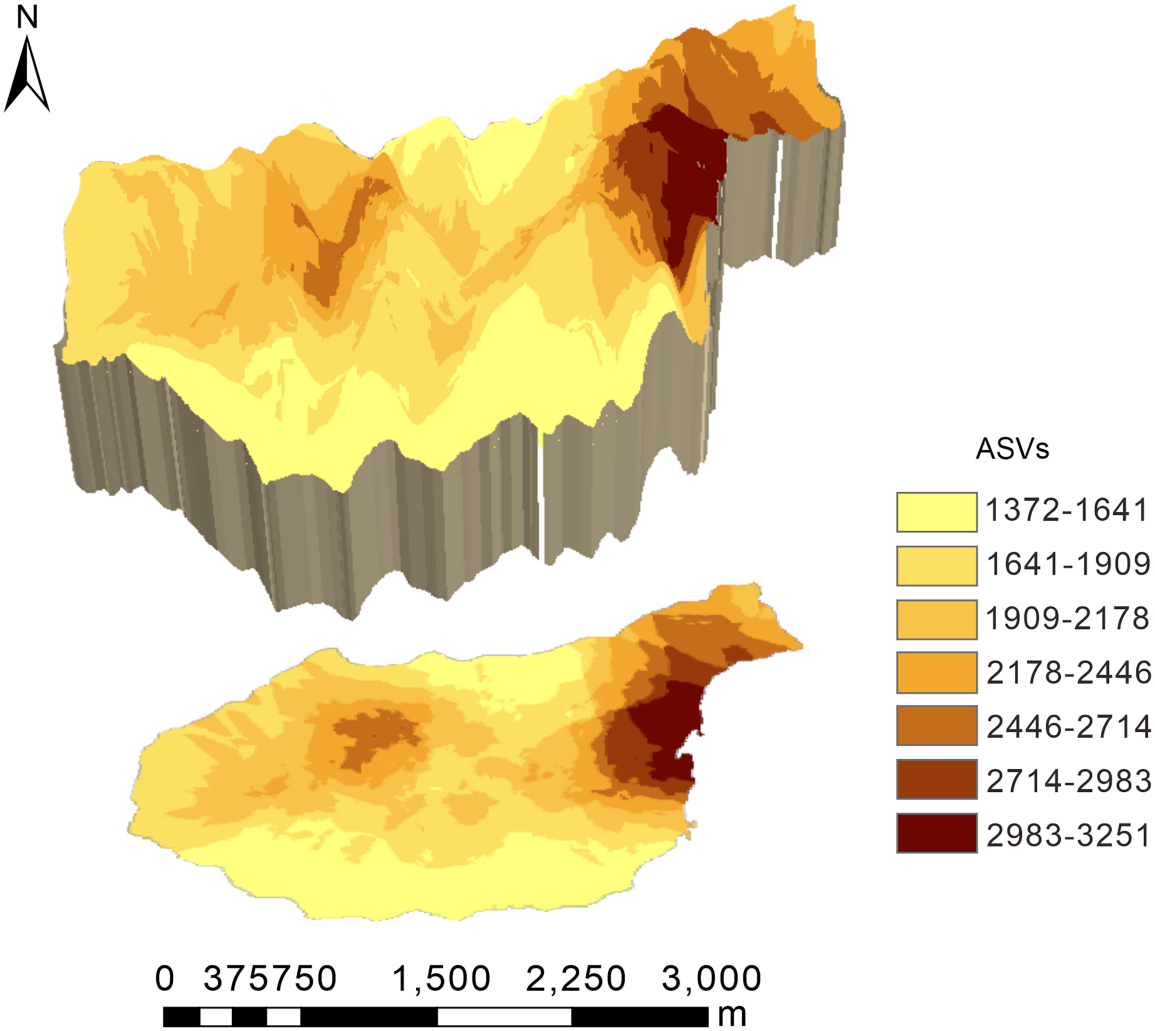
Spatial characteristics of relevant amplicon sequence variants (ASVs) in the Huoditang Forest in Qinling Mountains, China.

Original kriging interpolation was conducted to assess the spatial distribution map of the relative abundance of dominant bacterial phyla in the Huoditang Forest in the Qinling Mountains (Figure 3). The results showed that the spatial distribution patterns of the soil bacterial community components showed “peak,” “trough,” and “spot” patterns. The highest patch category of Proteobacteria occupied the largest area (2.56 km^2^), which was distributed in the southern part of the Huoditang Forest region (Figure 3). The highest patch categories of Gemmatimonadetes and Nitrospirae were also distributed in the south. Among the dominant taxa in the lowest patch categories, the habitat of Firmicutes occupied the largest area (11.93 km^2^). In addition, the relevant ASVs showed a decreasing “single peak” spatial distribution pattern, in which the ASV high-grade patches were distributed in the northeast of Huoditang Forest, accounting for approximately 4.82 km^2^ of patch area (Figure 4; Table 2).

### Links between soil bacterial taxa and vegetation factors, topographic factors, environmental factors, and soil nutrients

**Figure 5:**
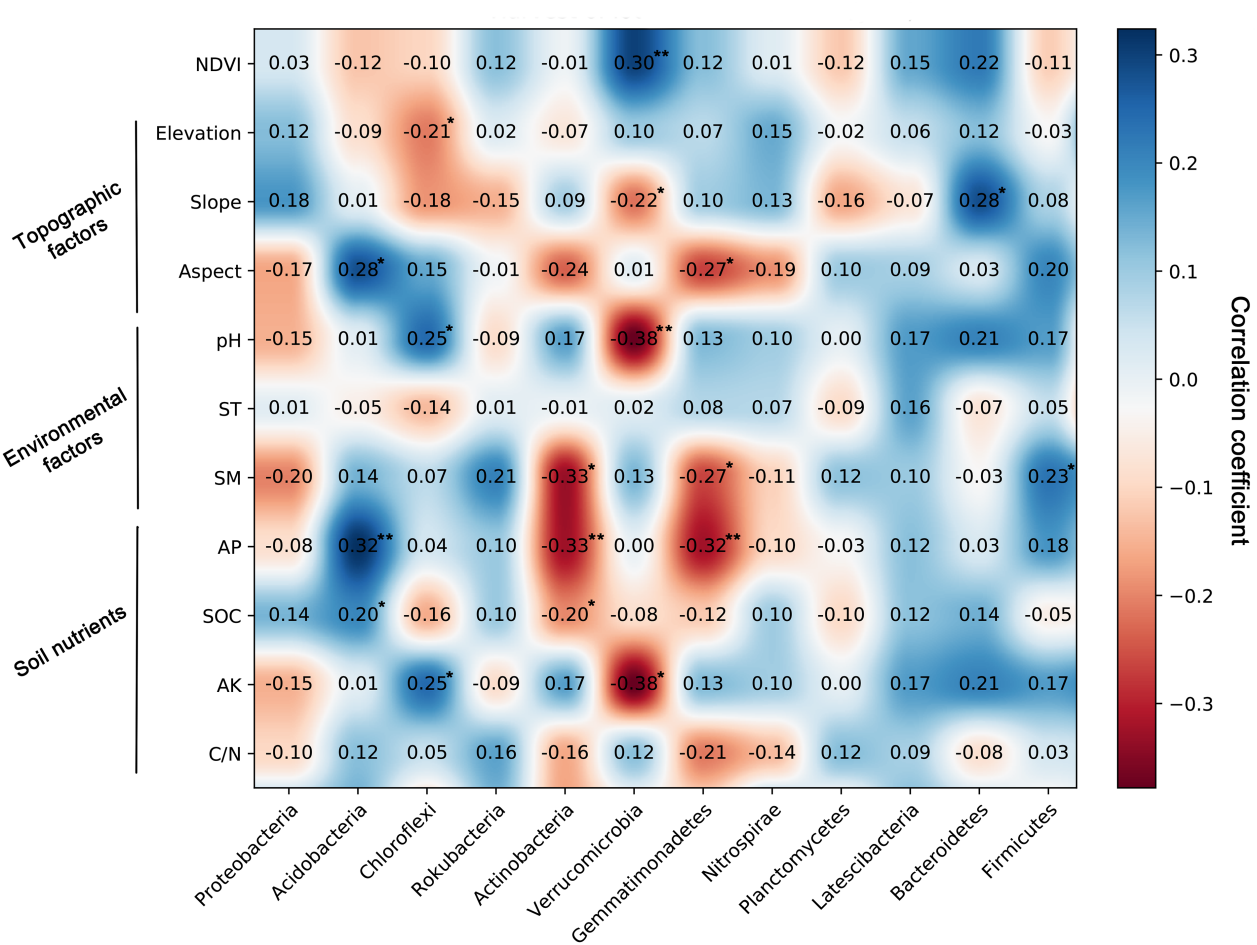
Spearman’s correlation coefficients between the relative abundance of bacterial communities and normalized difference vegetation index (NDVI), topographic factors, environment factors, and soil nutrients.

Spearman’s correlation analysis confirmed a significant relationship between soil bacterial community components and their influential factors (Figure 5). The relative abundance of Acidobacteria was significantly positively associated with the aspect (*r* = 0.28, *p* < 0.05), AP (*r* = 0.32, *p* < 0.01), and SOC (*r* = 0.20, *p* < 0.01), and Chloroflexi was effectively linked with elevation (*r* = −0.21, *p* < 0.05), pH (*r* = 0.25, *p* < 0.05), and AK (*r* = 0.25, *p* < 0.05). Moreover, the results showed that NDVI, slope, pH, and AK significantly affected Verrucomicrobia (Figure 5). The relative abundance of Actinobacteria was negatively significantly associated with SM (*r* = −0.33, *p* < 0.01) and the AP contents (*r* = −0.33, *p* < 0.01). Gemmatimonadetes was negatively related to the soil SM (*r* = −0.27, *p* < 0.05) and AP (*r* = −0.32, *p* < 0.01) content.

**Figure 6:**
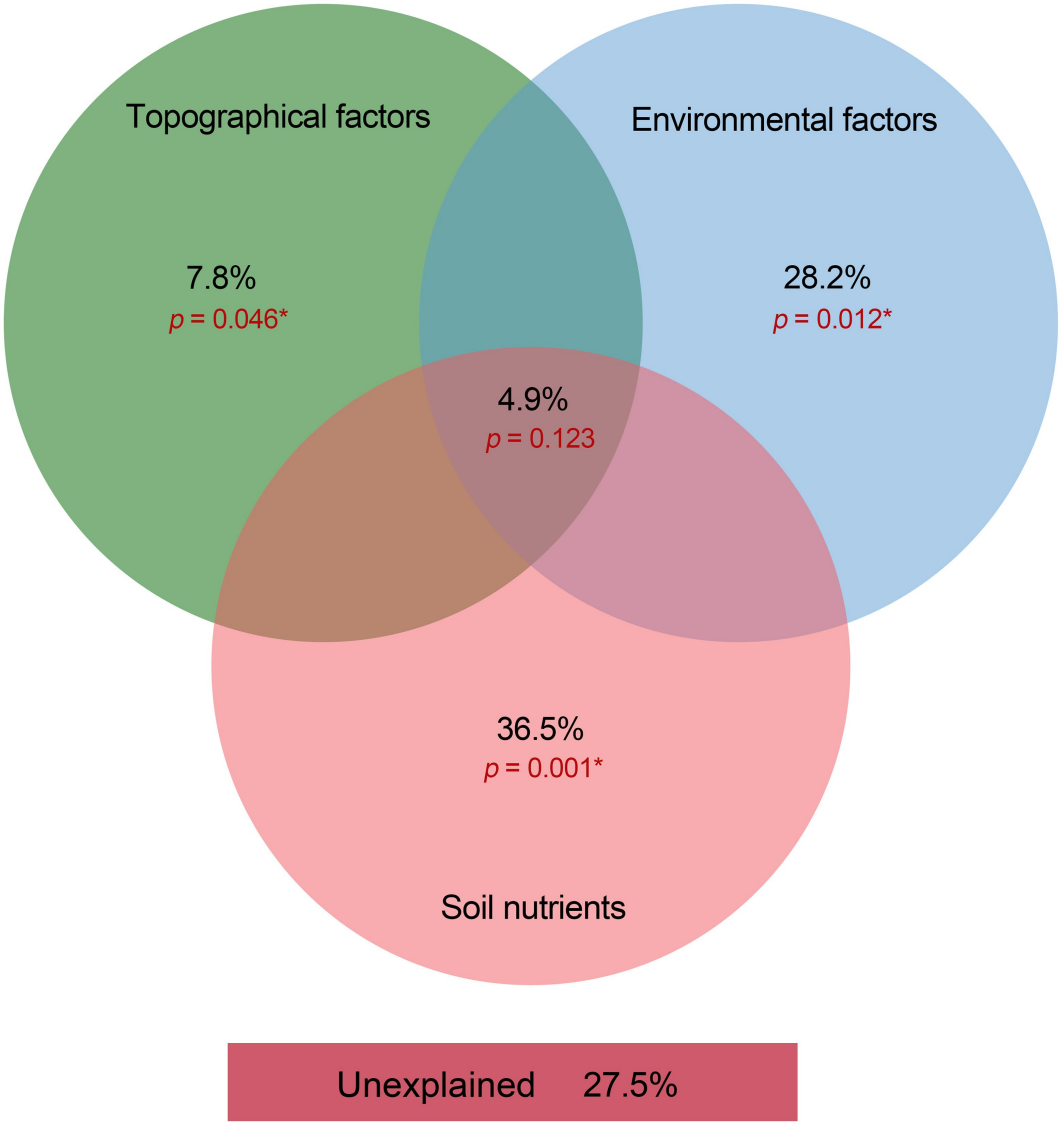
Variation partition analysis on the contribution of variance from topographical factors, environmental factors, and soil nutrients to the dominant bacterial phyla in the Huoditang Forest soils.

**Figure 7:**
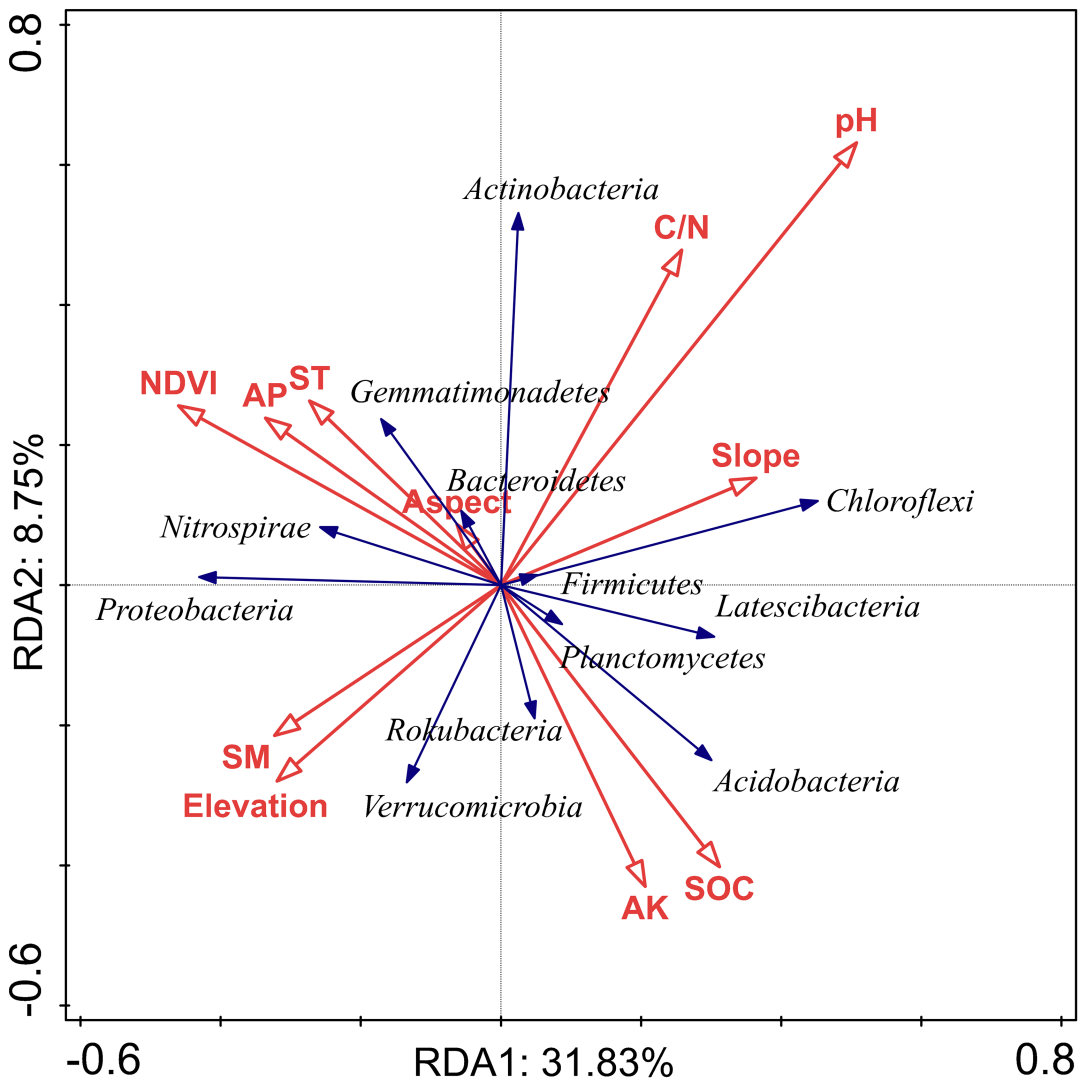
Redundancy analysis (RDA) between the relative abundance of the dominant bacterial phyla and environmental factors, topographical factors, and soil nutrients in Huoditang Forest region in Qinling Mountains, China.

We used VPA to clarify the relative contribution of bacterial community composition driven by topographical factors, local environmental factors, and soil nutrients (Figure 6). The VPA results indicated that topographical factors (*p* < 0.05), environmental factors (*p* < 0.01) and soil nutrients (*p* < 0.01) explained approximately 7.8, 28.2 and 36.5% of the bacterial community variation, respectively. This suggested that soil nutrients probably affect the spatial distribution of the bacterial community composition more than topographic and environmental factors in subtropical forest ecosystems. Moreover, topography, environment, and soil nutrients together accounted for 4.9% (*p* = 0.123) of the variation in the dominant bacterial taxa. The unexplained portion accounted for 27.5% (*p* < 0.01) of the total variation in the community (Figure 6). The RDA confirmed the Spearman’s correlation results and demonstrated the effects of topographical factors, environmental factors and soil nutrients on the dominant bacterial taxa. The RDA results revealed that NDVI, topographical factors, environmental factors, and soil nutrients accounted for 40.58% of the total variation (axes 1 and 2; Figure 7). Among the topographical factors, elevation was the most important driving factor and accounted for 3.9% of the variation (*p* < 0.01; Figure 7; Supplementary Table S2). As the representative parameters of the other two factors, pH, NDVI, SOC AP, and ST explained 9.1, 7, 6.6, 4.7, and 2.6% of the bacterial community variation (*p* < 0.05), respectively (Figure 7; Supplementary Table S2).

## Discussion

### Spatial patterns of the dominant bacterial taxa

The main bacterial community components were Proteobacteria (31.28%) and Acidobacteria (23.52%) in the Huoditang Forest region, which is consistent with previous studies on the North China Plain (Zhang et al., 2020), alpine steppe (Zhang et al., 2016), Loess Plateau (Liu et al., 2018), Mariana Trench (Cui et al., 2019), and Mt. Kilimanjaro (Shen et al., 2020). These areas have different climatic conditions and ecosystem types, indicating the dominance of core bacterial community components under different phenological conditions, which is crucial for a better understanding of the ecological effects of core taxa (Li et al., 2020). Compared with other dominant components, the highest patch category of Proteobacteria had the largest area and showed a “bimodal” pattern (Figure 3), which suggested that Proteobacteria were best adapted to the environment among prokaryotes (Yun et al., 2014). However, previous studies have shown that the soil core flora varies according to land use types, dominant tree species, and human activities (Urbanová et al., 2015; Naveed et al., 2016; Zhang et al., 2016). For example, the relative abundances of Acidobacteria in Canadian agricultural soil and subtropical mixed forest soil were both greater than that of Proteobacteria (Banerjee et al., 2016; Nie et al., 2021). This may be related to the pH of different land use types and forest types, as studies have found that Actinobacteria with aerobic metabolism had better growth in the pH range of 6.5–8.0 (Bhatti et al., 2017). In addition, the spatial heterogeneity of bacterial community components are probably associated with changes in the microtopography of mountain ecosystems, where microclimatic conditions alter certain factors (e.g., canopy cover and light and radiation intensity) that coerce microbial living space and create independent ecological niches (De Frenne et al., 2021). Notably, Zeng et al. (2019) observed that Acidobacteria tend to live in forest soils with relatively high SOC contents, which was supported by the results of this study that showed SOC was significantly positively related to Acidobacteria (Figure 5). In addition, Chloroflexi, Rokubacteria, Actinobacteria, Verrucomicrobia, Gemmatimonadetes, Nitrospirae, and Planctomycetes (relative abundance > 2%) were the dominant taxa, i.e., not Proteobacteria and Acidobacteria, and there were no continuous spatial distribution characteristics with the elevation gradient, which is similar to the results presented by Shen et al. (2020).

Strong spatial dependence was observed for the most dominant phyla based on GD values (< 20%), except for Gemmatimonadetes and Latescibacteria. The results also showed significant spatial autocorrelation (i.e., a “U”-shape) of dominant bacterial community components in a certain range at the regional scale, except for Actinobacteria and Verrucomicrobia (Figure 2; Table 3). Nevertheless, Song et al. (2018) found that the Moran’s *I* indices of Actinobacteria, Proteobacteria, and Firmicutes decreased with lag distance, while that of Thaumarchaeota was close to 0. These differences may be caused by differences in soil pH, vegetation types, and litter quality (Urbanová et al., 2015; Shen et al., 2020). Compared to the Qinling Mountains, karst forests in southwestern China have a neutral or slightly alkaline soil pH, resulting in a unique distribution pattern of core microbial community components (Song et al., 2018; Hu et al., 2020). Spearman’s correlation analysis confirmed that pH was significantly related to Actinobacteria (Figure 5). In addition, due to the trend of nitrogen saturation, the quality of litter in karst forests was higher than that in non-karst forests, which was one of the reasons for the existence of different spatial distribution patterns of dominant taxa (Liu et al., 2016). In any case, soil–plant–microbe interactions jointly determine above-ground and below-ground cycles in terrestrial ecosystems (Ding et al., 2021). For example, vegetation types have direct or indirect effects on the dominant bacterial phylum through litter production, root exudate production, and temperature and humidity changes (Prescott and Grayston, 2013). Thus, the unique ecological niche of soil microorganisms in the Huoditang Forest is probably influenced by a combination of vegetation type, topographic factors, and soil characteristics, resulting in unique spatial distribution patterns of different dominant bacterial community components.

### Relationships between soil bacterial community components and NDVI, topography, environment, and nutrients

Among the environmental factors, elevation was the most significant driving factor for the spatial heterogeneity of dominant bacterial community components (Figure 7). Previous studies have shown that the distribution of bacterial communities is characterized by “rising,” “falling,” “U,” and “single peaks” along elevation gradients (Yuan et al., 2014; Wang et al., 2015; Shen et al., 2020). Theoretically, soils located at high elevations are characterized by lower temperatures and less moisture, which severely limit the activity of microbial communities and put great pressure on their survival (Zhang et al., 2016). We found that there was a significant correlation between elevation and Chloroflexi (Figure 5), which indicated the niche differentiation of the horizontal spatial pattern of dominant bacterial taxa and the associated influence of the terrain (Figures 4, 7). However, the spatial heterogeneity of soil microbial communities was more indirectly influenced by elevation than by soil characteristics such as ST, SM, and SOC (Figure 6).

Previous studies have investigated the distribution of soil characteristics such as pH, AK, AP, C/N, SM, and SOC in the Huoditang Forest in the Qinling Mountains (Zhou et al., 2021). Among the environmental factors, pH played the most important role in driving bacterial community composition based on RDA in the Huoditang region of the Qinling Mountains (Figure 7; Supplementary Table S2). It has been widely confirmed that pH shapes the soil bacterial community and diversity in terrestrial ecosystems (Rousk et al., 2010; Shen et al., 2013; Zheng et al., 2019). Spearman’s correlation analysis also suggested that pH significantly affected the main dominant community components, including Chloroflexi, Verrucomicrobia (Figure 5), which was supported by the results of Zeng et al. (2019) for the Loess Plateau, Zhao et al. (2020) for saline agricultural soil, and Kang et al. (2021) for alpine wetlands. The possible explanation is that the spatial distribution of soil pH directly leads to the spatial characteristics of bacterial community components because the narrow range of pH variation at the regional scale may provide a suitable environment for bacterial growth (Cao et al., 2016). Another explanation is that soil pH directly affects the changes in other soil parameters (e.g., SOC and nutrient availability) and the vegetation community and further indirectly drives the variation in the soil bacterial community (Rousk et al., 2010; Urbanová et al., 2015; Zeng et al., 2019). For example, pH directly drives the decomposition of soil organic matter and indirectly affects the composition of the soil bacterial community in some ecosystem types (Djukic et al., 2010; Zeng et al., 2021). Here, we found that SOC was associated with dominant bacterial community components, which was also confirmed by VPA and RDA, which showed that soil nutrients (SOC) were a significant driver affecting the bacterial community components (Figures 6, 7). In general, the growth of vegetation in forest ecosystems is most affected by climatic conditions and soil nutrients, leading to differences in the SOC content along an elevation gradient and affecting the decomposition rate and functional diversity of soil microorganisms (Urbanová et al., 2015; Li et al., 2019). On the other hand, the relative abundance of Acidobacteria was significantly negatively correlated with SOC (Figure 5). Acidobacteria are oligotrophic bacteria (Fierer et al., 2007), they typically live in low-nutrient soil. We also found a significant correlation between SOC and Actinobacteria (Figure 5), as observed by Zhao et al. (2020) in agricultural soil and Zeng et al. (2019) on the Loess Plateau, indicating that SOC plays an indispensable role in shaping the soil bacterial community composition at the regional scale.

Our results provide evidence for the spatial pattern of the bacterial community in subtropical forest soil and its driving factors. However, how environmental conditions shape unique bacterial communities is still unknown at the regional spatial scale, especially in subtropical forests with high vegetation cover, complex climate, and high biodiversity. This is a limitation of this study and one of the directions that needs further research. This study can be used to design future studies investigating the functions of the soil fungal community in subtropical forests.

## Conclusion

Our results showed that Proteobacteria (31.28%), Acidobacteria (23.52%), Chloroflexi (13.39%), Rokubacteria (8.17%), Actinobacteria (7.47%), and Verrucomicrobia (5.36%) were the dominant bacterial community components. We found that dominant bacterial phyla, such as Proteobacteria, Acidobacteria, Chloroflexi, Gemmatimonadetes, and Nitrospirae, had special spatial heterogeneity and distribution characteristics on a regional scale. Meanwhile, the kriging interpolation showed that the dominant bacterial phylum did not show a “peak” distribution pattern but rather a “bimodal” or “multimodal” spatial pattern in Huoditang Forest soils in the Qinling Mountains. Among the driving variables, soil nutrients, environmental factors, and topographic factors explained 36.5, 28.2, and 7.8% of the total variations, respectively. Additionally, elevation, pH, AP, and SOC were considered significant driving factors for dominant bacterial taxa. These results add to the novel understanding and evidence using dominant bacterial taxa to provide predictions of spatial heterogeneity. The results can help predict the spatial distribution and influential factors of soil bacterial community compositions in typical subtropical forest ecosystems.

## Data availability statement

The raw data supporting the conclusions of this article will be made available by the authors according to the requirement of the researcher, without undue reservation.

## Author contributions

YoZ and XJ: conceptualization, supervision, project administration, funding acquisition, and writing—review and editing. YoZ, ML, and YuZ: methodology, investigation, and writing—original draft preparation. ML, QM, and YoZ: validation. ML, YuZ, and SY: formal analysis. ML, QM, and SY: data curation. ML, YuZ, QM, and SK: visualization. All authors contributed to the article and approved the submitted version.

## Funding

This work was jointly supported by the National Natural Science Foundation of China (No. 31670549), the Fundamental Research Funds for the Central Universities, CHD (300102351730, 300102270206, and 300102278403), and the Fund Project of Key Laboratory of Degraded and Unused Land Consolidation Engineering, the Ministry of Natural and Resources (SXDJ2019-03).

## Conflict of interest

The authors declare that the research was conducted in the absence of any commercial or financial relationships that could be construed as a potential conflict of interest.

## Publisher’s note

All claims expressed in this article are solely those of the authors and do not necessarily represent those of their affiliated organizations, or those of the publisher, the editors and the reviewers. Any product that may be evaluated in this article, or claim that may be made by its manufacturer, is not guaranteed or endorsed by the publisher.

## Footnotes

^1^ https://scihub.copernicus.eu/dhus/#/homepage

^2^ https://ccb.jhu.edu/software/FLASH/

^3^ http://qiime2.org/

^4^ http://www.arb-silva.de

^5^ https://matplotlib.org/
