# Supplementary material for: Spatial patterns of dominant bacterial community components and their influential factors in the southern Qinling Mountains, China
Source: Front Microbiol. 2022 Dec 23;13:1024236. doi: 10.3389/fmicb.2022.1024236 (PMC9816149; doi:10.3389/fmicb.2022.1024236)
Supplement: Supplementary file 1 [file Data_Sheet_1.doc]

**Table S1** Descriptive statistics of variable of the relative abundance of the dominant bacterial phyla.

| Species | Maximum (%) | Minimum (%) | Mean (%) | SE (%) | CV (%) | Skewness | Kurtosis |
| --- | --- | --- | --- | --- | --- | --- | --- |
| Proteobacteria | 72.80 | 10.00 | 31.28 | 9.80 | 31.34 | 0.04 | 0.81 |
| Acidobacteria | 42.80 | 6.70 | 23.52 | 6.22 | 26.43 | 0.55 | 0.92 |
| Chloroflexi | 38.90 | 2.80 | 13.39 | 6.71 | 50.12 | 1.32 | 1.83 |
| Rokubacteria | 17.30 | 1.30 | 8.17 | 2.96 | 36.19 | 0.12 | -0.07 |
| Actinobacteria | 36.20 | 2.30 | 7.47 | 4.18 | 55.92 | 3.68 | 20.41 |
| Verrucomicrobia | 22.00 | 0.20 | 5.36 | 4.52 | 84.28 | 1.23 | 1.08 |
| Gemmatimonadetes | 7.90 | 0.60 | 2.89 | 1.34 | 46.31 | 0.85 | 0.77 |
| Nitrospirae | 11.60 | 0.00 | 2.31 | 2.01 | 87.14 | 1.22 | 2.04 |
| Planctomycetes | 10.90 | 0.10 | 2.26 | 1.38 | 61.23 | 1.86 | 7.29 |
| Latescibacteria | 4.40 | 0.00 | 1.08 | 0.80 | 73.79 | 1.51 | 2.51 |
| Bacteroidetes | 5.50 | 0.00 | 1.01 | 0.67 | 66.87 | 2.26 | 10.23 |
| Firmicutes | 9.20 | 0.01 | 2.69 | 2.25 | 83.68 | 1.740 | 3.882 |

**Table S2** Results for RDA-test on the relationship between soil characteristics and the relative abundance of the dominant bacteria at phylum level in soils.

| Variables | | Phylum | |
| --- | --- | --- | --- |
|  |  | Explanation (%) | *p* |
| Topographical factors | Elevation | 3.9 | **0.014*** |
|  | Slope | 2.3 | **0.044*** |
|  | Aspect | ＜0.1 | 0.988 |
| Environmental factors | pH | 9.1 | **0.002**** |
|  | ST | 2.6 | **0.04*** |
|  | SM | 0.2 | 0.664 |
| Soil nutrients | AP | 4.7 | **0.006**** |
|  | SOC | 6.6 | **0.008**** |
|  | AK | 0.6 | 0.244 |
|  | C/N | 1 | 0.132 |
| NDVI | NDVI | 7 | **0.004**** |

ST, soil temperature; SM, soil moisture; AP, available phosphorus; SOC, soil organic carbon; AK, available [potassium](http://dict.youdao.com/w/eng/soil_available_potassium/#keyfrom=dict.phrase.wordgroup); C/N, soil C: N ratio.

***p* < 0.01;

**p*  <  0.05.
